# Project nGage: Network Supported HIV Care Engagement for Younger Black Men Who Have Sex with Men and Transgender Persons

**DOI:** 10.4172/2155-6113.1000236

**Published:** 2013-08-31

**Authors:** Alida Bouris, Dexter Voisin, Molly Pilloton, Natasha Flatt, Rebecca Eavou, Kischa Hampton, Lisa M Kuhns, Milton Eder, John A Schneider

**Affiliations:** 1 School of Social Service Administration, University of Chicago, Chicago, IL, USA; 2 STI/HIV Intervention Network, University of Chicago, Chicago, IL, USA; 3 Department of Medicine, University of Chicago, Chicago, IL, USA; 4 Access Community Health Network, Chicago, IL, USA; 5 Ann & Robert H. Lurie Children’s Hospital of Chicago, Chicago, IL, USA; 6 Department of Health Studies, University of Chicago, Chicago, IL, USA

**Keywords:** HIV/AIDS, Retention in care, Men who have sex with men, Transgender persons, African American, Adherence, Social support, Social networks, Network visualization, Information-Motivation-Behavioral skills model, Transtheoretical model

## Abstract

**Background:**

Young Black men who have sex with men and transgender persons (YBMSMT) aged 13–29 carry the nation’s highest burden of new HIV infections. Studies indicate that YBMSMT have poor retention in care, which is associated with reduced medication adherence and increased virologic failure.

**Objective:**

Project nGage is a randomized controlled (RCT) trial evaluating the feasibility and preliminary efficacy of a brief, dyadic intervention designed to promote adherence to HIV primary care in safety-net clinics. Network visualization is used to identify and engage a support confidant (SC) from participants’ social networks. A social work interventionist then meets with the SC and SC-participant dyad to activate and maintain HIV-specific social support.

**Methods:**

Project nGage is operating in two phases. In Phase I, the Team refined study protocols based on pilot testing. In Phase II, 94 HIV infected YBMSMT ages 16–29 will be recruited, enrolled and randomly assigned to receive Project nGage or treatment as usual (TAU). The primary outcome is appointment attendance; the secondary outcomes are medication adherence and viral load.

**Results:**

Implementation challenges include coordinating sites, managing dyadic intervention logistics, and recruiting non-adherent patients or those who have fallen out of care. The team continues to address implementation issues as the study progresses.

**Conclusions:**

Project nGage is addressing a gap in HIV care-related research by focusing on supportive relationships as a mechanism through which to promote retention in care. Pending study results, a larger RCT would compare the relative effectiveness of the Project nGage intervention versus TAU over 18 to 24 months.

## Introduction

From 2006–2009, HIV incidence among younger Black men who have sex with men (MSM) aged 13–29 increased by 48%; with HIV incidence unchanged among other young MSM [1]. In addition, epidemiological surveillance data show that transgender adolescents and young adults, especially transgender youth of color, are heavily impacted by HIV/AIDS [2,3]. Given high rates of HIV infection among young Black men who have sex with men and transgender persons (YBMSMT), engagement in HIV primary care, which includes *linkage to* and *retention in* care, is critical for reducing onward transmissions and outcome disparities [4]. Evidence suggests that virologic failure among Blacks occurs more often than Whites and is mediated by missed appointments [5,6]. Despite this link, there is a lack of empirically tested interventions designed for YBMSMT focused on linkage to care.

Given the importance of linkage to care for HIV-positive MSM, several existing strategies have sought to address engagement related challenges. The majority of such strategies are based on peer support groups [7], case management programs [8–10], multi-modal peer outreach [11,12], and peer health navigation [13] approaches. These efforts all share a common ingredient – newly created network members – a traditional public health intervention approach. In addition, other approaches such as providing financial incentives focus more on *linkage* than retention [14]. Although prior interventions have improved engagement in care [10,12], many programs require substantial resources on the parts of clinics and clinic staff, which may be not be available beyond the funding period. Multiple session interventions also require considerable commitment and effort on the part of program participants, many of whom may be contending with other demands on their time and resources. For example, in a study evaluating the efficacy of a case management intervention to engage and retain young Black and Latino MSM in HIV care, men were asked to attend weekly meetings with a case manager for two months before transitioning to monthly visits for the next 22 months [10]. In a separate study examining the effectiveness of multimodal programs to improve retention in care for African American and Latino MSM, case management was not associated with retention whereas attending a social support group was associated with better retention in HIV care one year later [12].

What is often missing from previous interventions is a coordinated attempt to harness organic, naturally existing social support networks in the lives of YBMSMT. Our use of “organic” refers to an endogenous existing social network, and can be contrasted with newly generated support persons, such as assigned peer navigators or lay health workers. The concept of organic social support as a powerful force in the health of HIV infected persons is well documented [15–18], but has been underutilized to retain YBMSMT in care. Living with HIV requires persistent, deep and ongoing social support [19]; support often only available from existing *confidants*– friends, partners, kin and other individuals – with whom one might share personal information with and be influenced by [20–23].

Project nGage was developed to capitalize on the empircally documented benefits of social supports [20,21] within the naturally organic networks in the lives of YBMSMT [24]. Unlike prior interventions, we identify, engage and intervene with a support confidant (SC), whose close relationship with index YBMSMT may promote adherence to HIV primary care. Whereas most dyadic interventions focus on a particular role, i.e., peer, parent, or partner, we use a flexible and patient-centered approach that selects SCs based on factors such as providing a supportive *function* (e.g., emotional support [25,26]) as opposed to their *status* (e.g., mother [27]). Such SCs are likely important not only for retention in care, but also for sustained health, risk reduction behavior maintenance and long-term adherence to antiretrovirals (ARVs) [28–30]. Furthermore, these organic systems are less dependent on external resources or staffing [31]. For example, our intervention is brief and relies on one face-to-face session with the SC and one joint session with the SC-index dyad, both completed in the same visit. Supplemental contact is conducted viabooster calls delivered via telephone or text messages. Brief interventions based on strong theories of behavior have demonstrated efficacy with numerous health risk behaviors, including sexual activity [27], alcohol use [32,33], and condom use [34]. Moreover, a systematic review of behavioral interventions to prevent HIV among MSM found greater reductions in unprotected anal intercourse in studies with interventions lasting one month or less [35]. Therefore, if nGage results are promising, this intervention may allow for longer sustainability.

### 

#### Theoretical background

**Figure 1:**
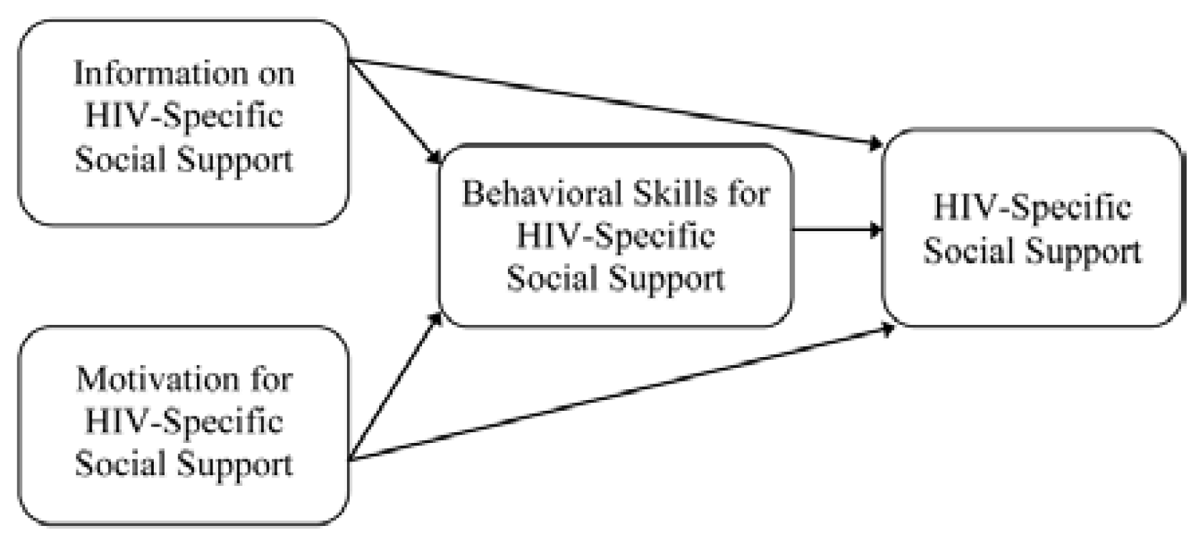
1MB Model for HIV Specific Support [32,53].

**Figure 2:**
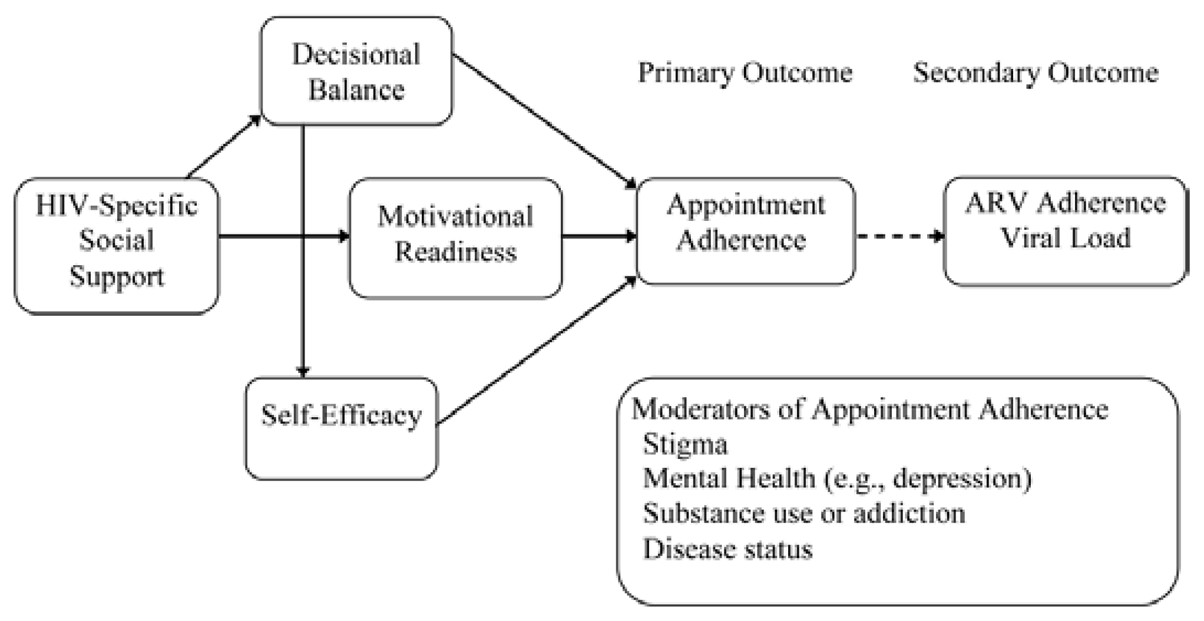
HIV Specific Support for Appointment Adherence [19].

The premise of Project nGageis based on theories of social support [36,37] and social networks [38]; together, these two theories are coupled with a network visualization approach [39] to help YBMSMT safely identify a SC to engage in retention in care activities. In addition, the content of the intervention is theoretically supported by the Information-Motivation-Behavioral Skills Model (IMB) and an adapted version of the Transtheoretical Model (Figures 1 and 2). While described separately, the two frameworks are intimately linked through their shared focus on HIV-specific social support within the dyadic relationship [27]. The first framework, IMB [40], is applied to the SCs to activate and maintain HIV-specific social support. This framework targets behavior at the *SC level* and allows us to focus on the factors that motivate HIV-specific social support on the part of SCs, namely information, motivation and relevant behavioral skills (e.g., self-efficacy and objective abilities). It also guides our decision to target motivation at the personal and social levels by addressing attitudes and beliefs about stigma and HIV-specific social support. The second framework is based on empirical applications of the Transtheoretical Model (TTM) that have been adapted to the relationship between social support and ARV adherence [19]. This allows for assessment and measurement of how HIV-specific social support from SCs at the *index YBMSMT level* affects the targeted behavior of appointment adherence.

Like IMB, adapted TTM posits that there are three determinants of behavior: (a) *motivational readiness* (i.e., how willing one is to engage in a behavior); (b) e*xpectancies* (i.e., the positive and negative outcome expectations one has when thinking about enacting a given behavior); and (c) *self-efficacy*. We posit that these three factors fully mediate the relationship between the receipt of HIV-specific social support from SCs and appointment adherence. By using this framework we can provide SCs with effective communication and behavioral strategies that target the motivational readiness, expectancies, and self-efficacy of YBMSMT.

### Study design and methods

Using a randomized controlled trial (RCT) design, 94 HIV infected YBMSMT ages 16–29 who have successfully been linked to care are randomized to an experimental arm or to a control arm consisting of treatment as usual (TAU). TAU is the standard of HIV primary care for this population and includes routine case management. During the pilot RCT, we seek to answer three key questions in preparation for our next step – an adequately powered RCT:

1. Is the proposed intervention acceptable and feasible?
2. To what extent did the intervention improve 12-month retention in care relative to TAU?
3. To what extent was there fidelity to the intervention?

Although determining the long-term impact of the intervention is beyond the scope of the pilot study, we are collecting important data on feasibility and initial efficacy. Specifically, we hypothesize that compared to participants in the TAU arm, YBMSMT in the intervention group will have fewer missed visits; increased perceptions of self-efficacy, dyadic closeness and social-support; greater adherence to ARVs; lower rates of unprotected anal intercourse; lower viral loads and higher intervention ratings. Pending current study results, design of a larger RCT would be designed to compare the relative effectiveness of the intervention versus TAU care over 18 to 24 months in HIV infected YBMSMT in safety net clinic settings on the South Side of Chicago. Institutional Review Board approval was obtained from the University of Chicago.

#### Phase I

Based on participant, staff, and interventionist feedback from pilot testing, core aspects of the nGage protocol were refined to include tailored feedback on YBMSMT’s existing social support network structure and a purposeful approach to select the most appropriate SC from the index’s personal network. Index participants are provided with a sociogram print-out of their social network, which serves as a vehicle to discuss power and relationship dynamics of social network members. Additionally, a single clinic visit for the SC-index dyad was included, consisting of an individual and a dyadic session. Pilot work suggested that prospective participants had limited availability and needed an intervention that was time-limited and maximized their mutual availability. Mini-booster sessions would be delivered via phone (to the SC) and text (to the index). HIV infected YBMSMT reported feeling overwhelmed by their diagnosis and the wealth of information provided during clinic visits; therefore, *mini-booster session* phone calls and interactive text messages delivered within pre-specified windows were planned. Finally, motivational interviewing (MI) [41] and problem-solving techniques from Cognitive Behavioral Therapy (CBT) [42,43] were integrated in order to quickly engage and help SC and index participants to address barriers to disclosure and behavior change.

It should be noted that our focus on the SC is purposeful in its intent to offload the intensity of appointments and intervention ingredients that typically target the index, even as part of standard TAU. Other programs might focus solely on the index participant for multiple sessions [44], yet such approaches may not be feasible or sustainable in safety net settings given the myriad of other case management, mental health screening, clinical testing/vaccination visits that clients who are newly infected or re-engaging in care require. Project nGage includes standard behavioral strategies that have been shown to improve the effectiveness of engagement in care interventions [27] (e.g., MI, CBT, problem-solving skills [33–35]) and unique social support network strategies developed from formative work [24,45–48] to help newly infected YBMSMT adhere to appointments and reduce HIV medication failure risk. Social support network strategies include approaches that utilize local structural features of an individual’s network to determine which network member may be uniquely positioned to provide support [49]. For example, a network member who is also tied to other network members who are unaware of the individual’s HIV status or risk behavior may not be as appropriate to an equivalently supportive individual who is not tied to such network members [50].

#### Phase II

We are in the process of enrolling 94 newly HIV infected YBMSMT ages 16–29 over a period of 18 months from two study sites. The two sites complement each other, as one is based in a university hospital and the other is a safety net clinic or federally qualified health center, which hosts university doctors. Although both sites provide services to an undeserved population, primary recruitment occurs at the safety net clinic.

#### Inclusion and exclusion criteria

Interested index participants are screened for eligibility, based on the following criteria: (a) Self-identify as African-American or Black, and male or transgender; (b) has had anal or oral sex with a man in the past 2 years; (c) English speaking; (d) has at least one potential SC in their network; (e) owns a cell phone not shared with other persons; (f) agreeable to text-message mini-booster sessions; (g) between the ages of 16–29 years old; and (h) HIV diagnosis for more than 3 months. Index participants are excluded if they are unable to provide assent/consent or plan to move out of the area within the next 12 months. Index participants who do not have at least one SC in their network (estimated to be less than 5%) [51] are referred to weekly group sessions for HIV infected clients.

SCs are also screened for eligibility based on the following criteria: (a) Index participant has agreed to engage this particular SC; (b) SC is willing to attend a face-to-face session and to receive booster sessions via telephone; (c) at least 18 years of age; (d) English speaking; and (e) owns a cell phone that is not shared with other persons. SC participants are excluded if they are unable to provide informed consent. We make sure that index and SC participants understand that screening for abuse (emotional, physical, sexual) occurs within the dyad, and they will be excluded from the study if either screen positive, with referral for additional psychosocial evaluation with partnering behavioral specialists, and other action if necessary.

#### Baseline visit

Eligible index participants undergo consent/assent and complete the baseline assessment within 30 days of being screened for eligibility. The baseline assessment includes survey modules on demographics [45], social support [25], substance use [52], mental health [53], dyadic closeness [54–56], stigma [57], self-efficacy [58,59], strain/abuse [60–62], sexual behaviors, social network characterization [51], and the constructs in Figures 1 [40,63] and 2 [19]. YBMSMT (and SC members recruited later in the experimental condition) are provided with $25 for completion of measures at baseline, 3 months and 12 months. In order to maximize comparability with other studies, we selected measures previously tested in studies of social support networks, particularly among youth, whenever possible.

#### Randomization and blinding

**Figure 3:**
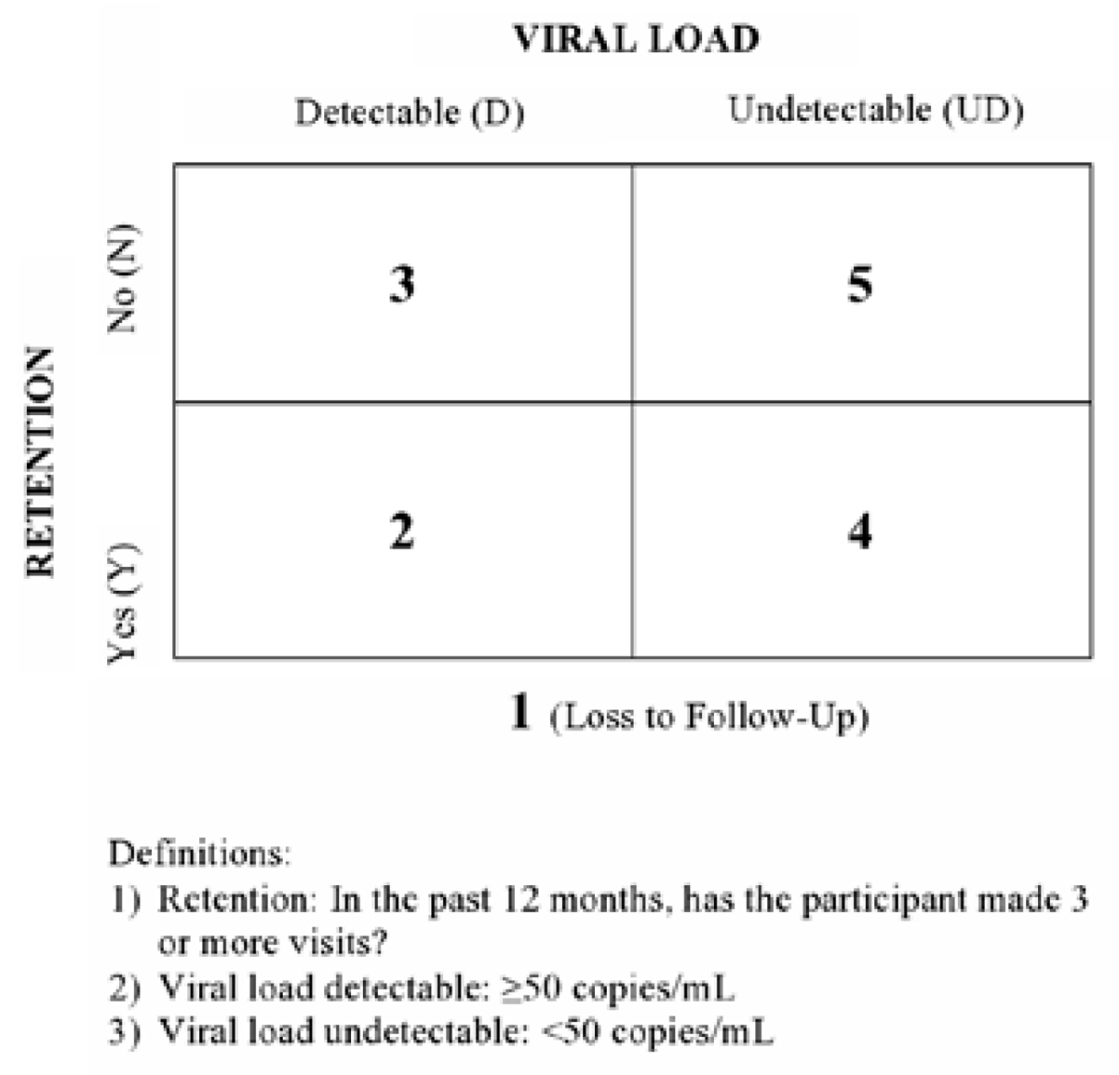
Randomization by Adherence and Retention Specturm.

Following the baseline assessment, study participants are randomized into one of two groups: the experimental condition or the TAU condition. Block randomization is used for allocation, which is further stratified by adherence and retention status to ensure balanced representation in the two treatment arms (TAU and intervention). Randomization across the adherence and retention spectrum is necessary to get a representative sample of the patients seen at both study sites, as well as for allowing further comparison across these groups. Before randomization occurs, patients are assigned to one of five groups based on their most recent viral load (i.e., < or > 50 copies per milliliter [ml] of blood) and the number of appointments they have attended in the last 12 months (i.e., did the patient attend at least 3 clinic visits in the past year?). Figure 3 illustrates the five categories for block allocation: (a) Block 1 is for patients who have not attended any clinic visits in the past 12 months, i.e., they have been lost to follow up; (b) Block 2 is for those who are retained in care (i.e., they have attended three clinic visits in the previous 12 months) and have a detectable viral load of > 50 copies/ml; (c) Block 3 is for patients who have attended less than three clinical visits and have a detectable viral load; (d) Block 4 is for YBMSMT who attended less than three clinical visits and have an undetectable viral load; and (e) Block 5 includes young men and transgender women who are retained in care and have an undetectable viral load (i.e., < 50 copies/ml).

Randomization assignment is generated using the R program [64]. Once randomized, each participant is given a study identification number and is allocated to their respective study condition. Due to the nature of this intervention, complete masking of participants, investigators, and interventionists is not possible. However, the social work interventionist (SWI) only has contact with the intervention group, and the providers and case managers responsible for TAU are not informed of the intervention assignment.

#### Support confidant (SC) recruitment

A major ingredient of Project nGage is the recruitment, engagement, and retention of SCs. During the index participant’s baseline visit, candidate SCs are determined by completing a sociogram of the index participant’s social network, which scores a candidate’s overall stability and consistency of support. The interventionist and the index participant review the sociogram results and discuss the index participant’s desired candidate. SCs are selected through this collaborative process, and are then contacted by the index participant, either in the presence of the SWI, or after hours using a study information sheet as a guide. Candidate SC participants are screened for eligibility, and consented during the first study visit. Regular review of the index participant-SC relationship is conducted by project staff and our abuse screener assesses for bidirectional emotional, physical and sexual abuse in the index-SC relationship. If the screener indicates abuse, the nGage team, which includes a child and adolescent psychiatrist and ethicist, discusses the case and determines whether to proceed with the intervention and/or remaining booster sessions. In cases where abuse is directed from SC to Index, the index has the opportunity to select a new SC and continue with the intervention. At any point during the 12 months, index can disengage the SC from the project without justification for why this decision is made. Participants are also offered referrals to agencies that specialize in interpersonal violence and victimization.

#### Intervention

The intervention occurs during a scheduled followup visit and consists of an individual session with the nominated SC and a joint session with the SC-index participant dyad. The joint session lasts for approximately 90 minutes and is the primary point of intervention. During this time, the SC is activated and motivated to provide HIV-specific social support to the index participant by the SWI. At the beginning of the session, the SWI meets with the SC and index for 20 minutes to discuss the important role that social support plays in the lives of YBMSMT living with HIV. Three types of social support are reviewed: (a) practical support, e.g., the tangible things that SCs can do for the index, such as lending money for a bus pass or providing a reminder about clinic appointments, (b) informational support, e.g., knowledge and skills related to living with HIV, and (c) emotional support, e.g., responding to index in an empathic and caring way, such as calling to check in, hanging out, or listening to problems and struggles. The SWI then engages the dyad in a focused conversation about the nature of their relationship, the importance of consistent or ongoing clinical care for the index, and how support from the SC might help the index to stay retained in care. This component of the intervention ensures that there is mutual understanding on the importance of social support and addresses the lived experience of social support in each dyad’s relationship.

Following this conversation, the SWI meets with the SC alone for 40 minutes to provide information on HIV/AIDS and the importance of appointment and medication adherence for helping YBMSMT to lead long and healthy lives. In addition, the SWI reviews common barriers to retention in care and adherence to ARVs, such as alcohol and other drug use, medication side effects, mental illness, and lack of transportation [4]. The SWI then works with the SC to identify potential barriers that may prevent them from supporting the index. After the barriers are identified, the SWI and SC identify and practice strategies for overcoming these barriers. This component also enables the SWI to identify specific areas where the SC may need additional support from Project nGage staff. Throughout the individual session, the SWI is specifically targeting the SC’s information, motivation, behavioral skills, and self-efficacy to provide effective HIV-related social support.

After the individual session, the SWI holds a final joint session with SC and index to develop a personalized “Care and Support Plan” [42,43]. This plan identifies the most likely barriers to staying engaged in care and selects the best solutions and strategies for each SC-index dyad. For example, transportation to the clinic may be an important barrier. In such cases, the SWI, index and SC work together to identify the best solution. i.e., borrowing $5.50 for bus fare to and from the clinic; calling the clinic’s case manager two weeks before the appointment to secure a bus pass; or the SC drives the index to and from the clinic. In other cases, substance use may operate as a barrier to taking ARVs or attending clinic visits on occasions when the index drinks alcohol or uses drugs; in such cases, strategies such as having the SC remind the index to take their ARVs before using substances, setting a reminder on a cell phone, or scheduling afternoon instead of morning appointments are all viable strategies. Whatever the solution, the collaborative approach enables each dyad to problem-solve around the specific issues they identify. In this way, the intervention is tailored to the needs of each index participant and the relationship dynamics of their selected SC.

After the intervention session, there are six and five brief booster sessions that are delivered to the SC via cellular telephone and to the index via text messaging, respectively. The booster sessions occur every other month during the course of the study. The booster sessions are delivered by the SWI and focus on two primary areas: (a) implementing the “Care and Support Plan” and (b) the status of the SC-index relationship. For the first area, the SWI asks each member of the dyad about appointment and medication adherence, and to assess the plan that was developed. If needed, the SWI can revisit the plan or help each member of the dyad to problem solve around support and adherence to care. For the latter area, the SWI asks a series of focused questions in order to determine whether the intervention has added any stress or strain to the relationship. Thus, while the boosters are primarily designed to reinforce key content, the booster protocol is flexible enough so that SWIs can provide additional, tailored support to each SC-index dyad.

## Addressing Implementation Challenges

As with any intervention study, the team has had to address a number of implementation issues. The first issue addressed by the team was to develop an equitable and sustainable community-university research partnership. On the university side, the team is composed of three faculty researchers representing medicine and social work, a project manager with a master’s degree in public health, and two interventionists with a master’s degree in social work (MSW). In turn, the community partners include a case manager (MSW), a clinical supervisor, and a PhD research director. The primary administrative, coordinating, and research supports are provided by the university partner, and the community partner assists with identifying and recruiting eligible YBMSMT. To maintain a strong relationship, the core team holds weekly meetings to discuss the study’s progress. All protocols, including recruitment, intervention and tracking of study participants, have been developed and reviewed by the team. In addition, the project manager attends larger staff meetings at each site to ensure that clinic staff is knowledgeable and supportive of the project. Although these meetings require considerable time and effort, regular communication amongst all partners has enabled the team to better integrate Project nGage into each clinic and to quickly identify and address problems in the field.

Coordinating the intervention across two clinical sites has posed logistical challenges, as each site has its own organizational culture and work flow. In response, the team elicited staff feedback to develop an implementation plan for each clinic. Central to each plan was the need to develop research protocols that do not interfere with the routine provision of clinical care. This is an ongoing challenge when conducting research in community settings [65], as agencies prioritize service delivery while researchers emphasize the development of knowledge [66]. Although regular project meetings have helped to develop and refine these protocols, the team has also grappled with how best to maximize finite staff and agency resources. Space is a premium in medical settings located in urban communities and Project nGage is no exception; both of the participating clinics provide primary and urgent care, as well as specialized HIV care, to underserved populations. Thus, while each work plan has been tailored to maximize available resources, the team must be flexible and responsive to the needs of a dynamic community setting.

Additionally, there are a number of unique challenges related to working with a dyad, rather than a single participant. First, project staff must quickly build a strong relationship based on empathy and genuine positive regard with both the index and SC. Without this type of rapport, it would be difficult to engage and retain YBMSMT and their SCs for the duration of the study. However, scheduling two people for one intervention appointment is a challenge, as both participants and SCs are busy. Open and flexible communication has been essential and will continue to play an important role. At the same time, this communication must be balanced so as not to over-burden participants with too much project contact, i.e., intervention session, boosters, and data collection appointment reminders. This communication must also be done in a way that establishes a clear boundary between the research study and regular clinic services.

Another challenge is recruiting individuals who are already non-adherent or who have fallen out of care. For those who are out of care, it is difficult to locate them and/or bring them back into care. Although project and clinic staff work together to locate prospective participants, this collaboration cannot interfere with the time and labor demands faced by clinic staff. This has added another layer of scheduling challenges for the project team. Some strategies have been implemented to overcome these challenges, including the team holding flexible schedules to be on-site where prospective participants are being seen by clinicians. Additionally, each clinic has set aside time and space for the team to recruit and meet with prospective participants. Moving forward, Project nGage will continue to recruit from both sites, following a projected recruitment schedule. It is our hope that recruitment will be successful based on the commitment and dedication of the project team, the collaboration between the two sites, and the ongoing adjustment of study protocols to meet project needs. The team will continue to learn lessons surrounding the project procedures and feasibility, and address further challenges as Project nGage continues to evolve.

Finally, a pervasive issue is the stress and stigma related to being Black, gay or transgender, and HIV positive [67–69]. Perceptions of HIV-related stigma can prevent many positive persons from being engaged and retained in care [70–72]. Therefore, HIV positive YBMSMT who have disclosed their status to at least one confidant may not represent all YBMSMT. Although our study enrollment numbers have been adequate, limiting recruitment efforts to primarily two sites would likely not be feasible for a larger RCT. Nevertheless, we are learning important lessons from this pilot which would help us scale up this intervention should it prove to be efficacious.

## Conclusions

Assisting HIV positive YBMSMT to remain in medical care is critical to implementing primary and secondary intervention approaches and to meeting the goals of the National HIV/AIDS Strategy [73]. Treatment as prevention is gaining widespread appeal in the international HIV/AIDS prevention community. Given that there are too few intervention models for YBMSMT and no known interventions that explore the efficacy of utilizing organic social supports to assist YBMSMT to remain engaged in primary care, the potential efficacy of Project nGage remains promising.
